# Changes in psychiatric admissions to psychiatric wards due to COVID-19

**DOI:** 10.1192/j.eurpsy.2023.1243

**Published:** 2023-07-19

**Authors:** O. Martin-Santiago, M. C. Vallecillo-Adame, T. Jimenez-Aparicio, A. Perez-Escudero

**Affiliations:** 1 Department of Psychiatry, Hospital Clinico Universitario de Valladolid, Valladolid; 2 Complejo asistencial de Zamora, Zamora, Spain

## Abstract

**Introduction:**

Functioning in acute inpatient psychiatric units has been challenged by the Coronavirus disease 2019 (COVID-19). Patients with more severe mental health symptoms changed their rates of voluntary admission to psychiatric wards during the onset of the pandemic. Peritraumatic distress scores and increased likelihood of being psychologically affected by the COVID-19 pandemic can lead to a psychiatric admission. However, other factors could prevent hospitalization.

**Objectives:**

The present investigation aimed at admission rates of patients by depression, adjustment disorder or suicidal behaviour to a General Hospital Psychiatric Ward. We compared the lockdown due to COVID-19 in 2020 to similar periods of 2018 and 2019.

**Methods:**

The data of one general hospital psychiatric ward admissions have been obtained and analysed. We compared admission characteristics of 237 patients between April and June of 2018 and 2019 with 79 patients in the same period of 2020 (lockdown).

**Results:**

During the COVID-19 lockdown, there was a 35.8% reduction in psychiatric admissions and a significant reduction in psychiatric admission was observed due to suicidal behaviour (IRR = 0.49; 95% CI: 0.26-0.89; p=0.002) and depression (IRR = 0.24; 95% CI: 0.08-0.68; p=0.007), but similar rates of adjustment disorders (IRR = 1.12; 95% CI: 0.58-2.15; p=0.73).

**Conclusions:**

We suggest that patients with depression or suicidal behaviour avoided admission, for fear of contagion in hospitals. Also, greater family support could prevent self-injurious behaviours. By contrast, other disorders continued to require the same admission rate, since the social consequences derived from confinement could lead to the genesis or worsening of symptoms, such as adjustment disorders.

**Disclosure of Interest:**

None Declared

